# IAOseq: inferring abundance of overlapping genes using RNA-seq data

**DOI:** 10.1186/1471-2105-16-S1-S3

**Published:** 2015-01-21

**Authors:** Hong Sun, Shuang Yang, Liangliang Tun, Yixue Li

**Affiliations:** 1 School of Life Science and Technology, Tongji University, Shanghai 200092, China; 2 Key Laboratory of Systems Biology, Shanghai Institutes for Biological Sciences, Chinese Academy of Sciences, Shanghai 200031, China; 3 Shanghai Center for Bioinformation Technology, Shanghai 200235, China

**Keywords:** Overlapping transcription, High-throughput RNA-seq data, estimation of expression levels

## Abstract

**Background:**

Overlapping transcription constitutes a common mechanism for regulating gene expression. A major limitation of the overlapping transcription assays is the lack of high throughput expression data.

**Results:**

We developed a new tool (IAOseq) that is based on reads distributions along the transcribed regions to identify the expression levels of overlapping genes from standard RNA-seq data. Compared with five commonly used quantification methods, IAOseq showed better performance in the estimation accuracy of overlapping transcription levels. For the same strand overlapping transcription, currently existing high-throughput methods are rarely available to distinguish which strand was present in the original mRNA template. The IAOseq results showed that the commonly used methods gave an average of 1.6 fold overestimation of the expression levels of same strand overlapping genes.

**Conclusions:**

This work provides a useful tool for mining overlapping transcription levels from standard RNA-seq libraries. IAOseq could be used to help us understand the complex regulatory mechanism mediated by overlapping transcripts. IAOseq is freely available at http://lifecenter.sgst.cn/main/en/IAO_seq.jsp.

## Background

The advent of genome-wide techniques for studying transcription has strongly indicated that the majority of the genome can be transcribed [1-3]. Genome-wide overlapping transcription has been reported in various animal and plant species [4-9]. Multifunctional usage of the same genomic space leads to identical cDNA sequences produced from the same or opposite strands of DNA. The overlapping regions can include the exons in mRNAs, and a large number of transcripts from overlapping genes do not encode proteins [10-13]. Overlapping transcription is a highly conserved phenomenon that spans the animal, plant and fungal kingdoms, constituting a common mechanism for regulating gene expression.

The overlap of sense-antisense gene pairs can affect the regulation of gene expression at several levels including transcription, messenger RNA processing, splicing, stability, cellular transport and translation [14-16]. Natural antisense transcripts (NATs) are frequently functional and use diverse transcriptional and post-transcriptional gene regulatory mechanisms to carry out a wide variety of biological roles. Given the diverse regulatory functions and the widespread abundance of NATs in the human genome, it is not a surprise when some NATs were implicated in human diseases. Studies have shown that changes in antisense transcription were implicated in pathogenesis [17-19], indicating that activated antisense transcripts might be potential molecular markers for disease risk, as well as serving as novel therapeutic targets. However, apart from a few experimentally validated cases, the physiological roles of antisense transcription and the underlying mechanisms are largely unknown.

In-depth analysis of the transcriptome of overlapping genes is a valuable way for understanding the overlapping transcripts-mediated regulatory mechanism. A major limitation to the development of overlapping transcripts assays is the lack of high throughput expression data. Expression profiles of antisense and their sense targets can be used to infer the regulatory mechanism of action and the mechanism of antisense function. Techniques, like serial analysis of gene expression (SAGE) and cap analysis gene expression (CAGE), have been extensively used for the analysis of overlapping transcription [20,21]. Both of these methods have disadvantages and are much expensive to perform [22]. The widely used high-throughput microarray method, when dealing with probes mapped to the overlapping regions of same-strand overlapping genes, would provide no help to distinguishing signals from the original mRNA templates.

Next generation sequencing as a powerful tool has made dramatic improvement in sequencing cDNA derived from cellular RNA in a massively parallel and cost-effective way [23]. Recently developed techniques lead to more efficient assembly of individual transcriptomes. TIF-Seq determine both transcript ends by jointly sequencing the 5' and 3' ends of each RNA molecule [24]. RNA paired-end tags (RNA-PET) could demarcate the genomic boundaries of PET-represented DNA fragments [25]. However, standard libraries for RNA-seq, the most commonly used protocol, do not preserve information about which strand was originally transcribed, and strand specific RNA-seq method is labor intensive and requires substantial amounts of starting material [26,27]. Furthermore, though strand specific library construction preserves information about the orientation of transcripts, most current studies analyzed cDNAs without strand information because of its inefficiency and artifacts of reverse transcription.

Several methods have developed to reconstruct novel transcripts [28], and estimate isoforms abundances [29]. There are also several bioinformatics methods developed to infer strand information from non-strand specific RNA-seq data based on information such as open reading frame (ORF) in protein coding genes, biases in coverage between 5' and 3' ends or splice site orientation in eukaryotic genomes [30-32]. However, when dealing with reads mapped within exon challenge must be overcome to the inference without splicing information; besides, for those reads mapped within overlapping regions of same strand overlapping genes, even strand specific RNA-seq methods could not distinguish which strand was present in the original mRNA template.

To solve these problems, we developed a new method, IAOseq, to infer abundance of overlapping genes from high-throughput RNA-Seq data constructed by standard library. Levin *et.al*. had built a compendium of yeast libraries using several strand specific protocols and a non-strand specific protocol under same biological condition [26], which makes it possible to verify the performance of IAOseq. We therefore applied our method on the non-strand specific RNA-seq dataset (nonST in short) to infer expression levels of overlapping genes and use the strand specific dataset to test the validity of the method. Compared with other five most commonly used quantification methods, IAOseq yielded much better inferences.

## Methods

According to the yeast genome annotation, about eighteen percent of yeast genes are overlapping genes, most of which are located on different strand and about one-fifth are multi-gene overlaps (Additional file 1: Table S2). The average overlapping length is 290 bp for yeast overlapping genes (Additional file 1: Figure S1), and in mammalian genomes it is longer than 1 Kb [3]. Sequence reads obtained from the common next generation sequencing platforms, including Illumina, SOLiD and 454, are often very short (30-400 nt) [27]. Therefore, there is a high possibility that reads, which are shorter than the overlapping length, would be fully mapped to the overlapping regions with the result that strand information cannot be inferred by subsequent computational analyses using informations such as splice site orientation *etc*, leading to an overestimation of overlapping genes' expression levels.

### Implementation of IAOseq

**Figure 1:**
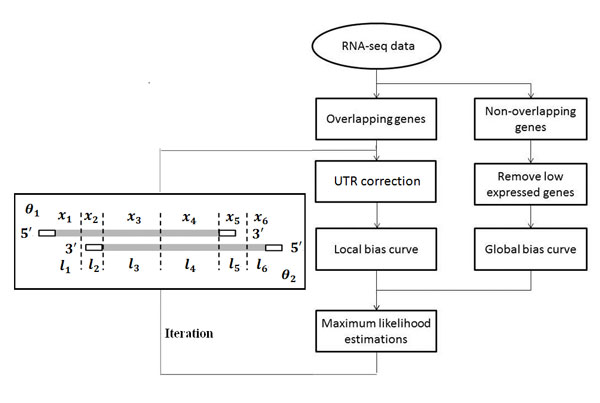
**Flowchart of IAOseq**.

To address this issue, we firstly divide annotated genes into two categories according to their genomic locations: overlapping genes and non-overlapping genes. To accurately infer overlapping genes' expression levels from nonST data, the overlapping regions are further divided into sub-regions as illustrated in the left box of Figure 1. Assume a transcribed genomic region contains m overlapping genes with expression levels $\left(\theta_{1},....,\theta_{m}\right)$. The transcribed region is split into n sub-regions with length $\left(l_{1},....,l_{n}\right)$ based on the overlapping pattern. A set of read counts $\left(x_{1},....,x_{n}\right)$are got from nonST data, where $x_{j}$ is the total read counts mapped to the *j*-th sub-region. An indicator matrix ${\left(a_{ij}\right)}_{mxn}$ is introduced to describe the overlapping pattern of the transcribed region, where $a_{ij}=1$ or $a_{ij}=0$ indicates whether the *j-*th sub-region is included in or excluded from the *i*-th gene respectively.

Under the assumption that sequenced reads are sampled independently and uniformly, according to the Poisson distribution model proposed by Jiang *et al*. when modeling the distribution of an individual sample [33], the read counts $x_{j}$ would follow a Poisson distribution with parameter $\lambda_{j}$, and $\lambda_{j}=l_{j}w\overset{m}{\underset{i=1}{\sum }}a_{ij}\theta_{i}$, where *w *is the total number of mapped reads. As reads distributions in most RNA-Seq datasets are not uniform [34], two bias curves, the global bias curve (GBC) and the local bias curve (LBC) are introduced to revise the indicator matrix $a_{ij}$. The GBC represents the general tendency of reads distribution for the whole transcriptome, and the LBC depicts gene-specific read distribution [35].

GBC is constructed from the non-overlapping gene sets because of its independence on specific genes. Reads distribution of a genomic region covered by overlapping genes is a mixture distribution of all its expressed genes. LBC is thus constructed to approximately describe the trend of read distribution along each gene [35]. For regions covered by overlapping genes, a step function is introduced for each gene on the *j*-th transcribed sub-region as $x_{j}/\left(l_{j}\overset{m}{\underset{i=1}{\sum }}\theta_{i}a_{ij}\right)$, j = 1,2,...n, which means the read counts are normalized by the sub-region length and the gene occurrences, and the read counts are weighted by expression level. The LBC of the gene is further got by normalizing the step function to be of mean 1.

A weighted indicator matrix $G_{ij}$ is got from GBC. The non-zero elements in $G_{ij}$ are weighted by the expression level of the *j*-th transcribed sub-region of the *i*-th gene. In the same way, a weighted indicator matrix $L_{ij}$is got from LBC. The two weighted indicator matrix $G_{ij}$ and $L_{ij}$ are combined together as $b_{ij}=\alpha \left(G_{ij}\right)+\left(1-\alpha \right)L_{ij}$ to take the place of $a_{ij}$ in order to revise the parameter $\lambda_{j}$ in the Poisson distribution function. In this study, α is set to 0.1 (Additional file 1: Note and Table S1).

For a transcribed sub-region that has $x_{j}$ reads mapped, the corresponding likelihood function is defined as $L\left(\Theta |x_{j}\right)=\frac{e^{-\lambda_{j}}{\lambda_{j}}^{x_{j}}}{x_{j}!}$.

Assume the read counts of each transcribed region are independent from each other, the joint log-likelihood function for gene members of the overlapping group is $\text{log}\left(L\left(\Theta |x_{1},....,x_{n}\right)\right)=\overset{n}{\underset{j=1}{\sum }}\text{log}\left(\frac{e^{-\lambda_{j}}{\lambda_{j}}^{x_{j}}}{x_{j}!}\right)$

Then, we have

$$\text{log}\left(L\left(\Theta |x_{1},....,x_{n}\right)\right)=-w\overset{n}{\underset{j=1}{\sum }}\overset{m}{\underset{i=1}{\sum }}l_{j}b_{ij}\theta_{i}+\overset{n}{\underset{j=1}{\sum }}x_{j}\text{log}\left(l_{j}w\overset{m}{\underset{i=1}{\sum }}b_{ij}\theta_{i}\right)-\overset{n}{\underset{j=1}{\sum }}\text{log}\left(x_{j}!\right)$$

Due to the convexity of the function, the gradient descending method is used to compute the maximum likelihood estimator Θ [33], that is, the expression levels of overlapping genes. We set initial value 1 to $\theta_{i}$ and iterate the optimization process, the $\theta_{i}$ is updated after each iteration process. Figure 1 illustrates the flowchart of the method.

### Correction of reads count in UTRs

Most overlapping regions involve UTR, therefore, it's necessary to include the UTR region for the overlapping analysis since UTRs are important parts of the transcript sequence. Alternative polyadenylation and transcriptional start sites could result in mRNA isoforms with variations in their untranslated regions, reads counts in UTRs are thus corrected according to a general tendency learned from reads distribution in UTRs of non-overlapping genes.

As reads distributions are not uniform, bias curve UTR(z) is introduced to revise the estimation of reads in UTR. To simulate the general tendency of reads distribution along UTR, UTR(z) is constructed from those non-overlapping gene sets without intersection with any other gene body or extended UTR. Assume the non-overlapping dataset contains t genes (p_1_, p_2_... p_t_). The normalized general tendency of reads number mapped to the *z*-th nucleotide is defined as,

$$UTR\left(z\right)=\left(\overset{t}{\underset{c=1}{\sum }}\frac{depth_{p_{c}}\left(z\right)}{depth_{p_{c}}\left(0\right)}\right)/t,$$

where *z *stands for the *z*-th nucleotide from the nearest coding nucleotide and *depth*(*z*) is the number of reads mapped to it.

The median lengths of yeast UTRs were estimated to be around 50 bp for 5'UTR, and 100 bp for 3'UTR [36]. Coding regions of yeast genes are therefore extended to 200 bp for 3'UTR correction and 100 bp for 5'UTR correction. The corrected reads count $x_{j}^{\prime }$ for the extended UTR region of the *i*-th overlapping gene is estimated as,

$$x_{j}^{\prime }=x_{j}-\overset{UTRlength}{\underset{z=1}{\sum }}UTR\left(z\right)depth_{\theta_{i}}\left(0\right)$$

The reads count in UTRs is replaced by $x_{j}^{\prime }$ in the above log-likelihood function.

### Data

#### RNA-seq datasets

Currently, qRT-PCR appears to be the most popular technology for producing "gold standard" abundance measurements; however, there is limit to get qRT-PCR results of genes enough for the overlapping analysis from public datasets, and it is also difficult to get RNA-seq datasets under the same biological condition. Levin *et.al*. built a compendium of yeast libraries using several strand specific protocols and a non-strand specific protocol, and sequenced them to deep coverage [26]. All these libraries were constructed under the same biological condition. Comparisons of the performance between these libraries showed that the dUTP second strand marking method (dUTP in short) performed reasonably and had the best quality measures of the strand specificity [26]. Therefore we applied our method on the nonST data to infer expression levels of overlapping genes and used the dUTP dataset to test the validity of the method. All sequencing reads in fastq format were aligned to the yeast reference genome using Bowtie software [37]. RSEM program [38] was used to deal with multiple mappings, and the posterior probabilities assigned were taken into account when estimating transcript abundance.

#### Simulated RNA-seq dataset

As there is few expression data for overlapping genes, we performed simulation experiments to further study the performance of IAOseq. UTRs are important parts of the transcribed sequences; we therefore extend all the annotated yeast gene loci 250nt on both sides. RSEM program [38] was used to generate a set of 1.3 million RNA-Seq fragments in a non-strand specific manner from the yeast transcriptome. The expression levels estimated from dUTP data are taken as input abundance estimates, and sequencing model parameters are set same as those obtained from nonST data.

### Gene annotations

Yeast genome annotations were downloaded from SGD database. SGD classifies yeast ORFs into three categories: verified, uncharacterized and dubious ORFs [39]. Though dubious ORFs are unlikely to encode a protein [39], we observed expression evidence for some of them from the dUTP data (Additional file 1: Figure S2). Furthermore, many ORFs classified as "dubious" overlap with ORFs of the class "verified" or "uncharacterized", we therefore used all annotated genes to test the method in this study. Of the overlapping groups analyzed in this study, forty-seven groups contain non-coding genes.

All the data were converted into a common version for comparison. The annotated yeast transcribed regions were classified into two categories: regions covered by overlapping genes and regions comprising only one single gene. Those transcribed regions of overlapping genes were further split into parts based on their overlapping patterns.

## Results

There are two principal types of overlapping transcripts: the same strand overlapping type in which the genes involved are transcribed from the same strand, and the different strand overlapping type in which at least two genes are transcribed from different strands [3]. Of the overlapping genes in yeast genome, around 76% are different strand type (Additional file 1: Table S2).

As strand specific RNA-seq could not distinguish transcripts from same strand overlapping genes, we therefore tested our method on two overlapping genes transcribed from different strand in the first place, then applied the method to the inference of expression levels of same strand overlapping genes, and then to the multi-overlapping genes constituted by more than two overlapping genes with a mixture of overlapping types. Short overlapping regions, where reads are much longer and would be mapped to the overlapping junctions, have little impact on the inference of strand information. IAOseq was thus trained on overlapping genes with overlapping length greater than 150bp.

Expression levels are measured in fragments per kilobase of exon model per million mapped reads (FPKM). The logarithm base 2 of estimated abundance ratio (LEARatio in short) was introduced as a measure to evaluate the performance, which is based on the expression level deduced from nonST data divided by the expression level from dUTP data. The LEARatio close to zero reflects the more accurate inference. To evaluate IAOseq, we compared its performance to five other commonly used quantification methods, *i.e*. Cufflinks [30], Isoem [40], RSEM [38], eXpress [41] and Bitseq [42]. As small difference was observed between values inferred using Isoem and using RSEM (data not shown), average abundance over the values estimated by the four methods (Cufflinks, RSEM, eXpress and Bitseq) from dUTP data was used as the denominator of the LEARatio.

### Application on real RNA-seq data

We first applied the five commonly used methods to estimate transcript abundances, and compared the expression level deduced from the nonST data with that deduced from the dUTP data. The scatter plots showed two distinct pattern, with a group of dots concentrated around the diagonal and another group of points scattered around the left-vertical line (Additional file 1: Figure S3), indicating a strong overestimation of expression levels especially for those genes with relatively low transcription levels. Estimating expression levels of lowly expressed genes would be much more affected by the inclusion of reads transcribed from the opposite strand.

**Figure 2:**
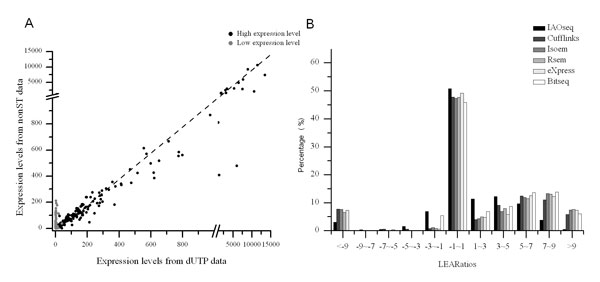
**Performances on different strand overlapping genes**. Scatterplot of the expression levels estimated using IAOseq from nonST data and the average expression levels using other four methods from dUTP data (A), and percentage of genes within LEARatio intervals for the comparison between IAOseq and the five commonly used methods (B).

In contrast with the five methods, IAOseq greatly reduced overestimation of transcription levels for lowly transcribed overlapping genes (Figure 2A). Considering correlation between expression levels deduced from nonST and dUTP data, we got a square of correlation coefficient of 0.61 using IAOseq, which is much greater than that by other five methods (Additional file 1: Table S3).

Compared with the five widely used methods, LEARatios of IAOseq were mostly concentrated in a narrow range close to zero with significantly lower standard deviation (Table 1 Figure 2B). IAOseq significantly reduced the overestimation of expression levels affected by the inclusion of reads transcribed from the opposite strand. Around 37% of overlapping genes are overestimated more than two-fold using IAOseq, which is much less compared with other four methods, where the percentage of genes with more than two-fold overestimation is 43% using Cufflinks, 47% using RSEM, 43% using eXpress and 48% using Bitseq. The results indicated validity of our method in the improvement of RNA-seq data analysis

**Table 1:** Summary of LEARatios for the IAOseq and the five commonly used quantification methods performed on different strand overlapping genes.

	Mean	Median	Standard deviation	P value (Wilcoxon test)	P value (Ansari-Bradley test)
IAOseq	0.53	0.05	5.70	---	---

Cufflinks	1.87	0.13	5.50	2.3e-08	3.6e-03

Isoem	3.22	0.65	3.70	2.2e-16	2.1e-09

RSEM	3.21	0.69	3.64	2.2e-16	9.8e-10

eXpress	-0.13	0.33	14.4	2.2e-16	4.2e-03

Bitseq	2.80	0.53	3.68	2.6e-10	1.9e-07

To test the significance of performance difference between IAOseq and the five commonly used quantification methods, we used Wilcoxon rank test for the median difference and Ansari-Bradley two-sample test for the variance difference of LEARatios.

### Application on same strand overlapping genes

In yeast genome, more than three hundred genes have same strand overlapping transcripts (Additional file 1: Table S2). When dealing with transcription signals mapped to the overlapping regions of the same strand overlapping gene pairs, most commonly used high-throughput methods for measuring gene expression, *i.e*. microarray or strand specific RNA-seq, could rarely distinguish which strand was present in the original mRNA template. Our proposed computational pipeline is not restricted to the overlapping types and can be applied to correct expression levels of same strand overlapping genes.

**Figure 3:**
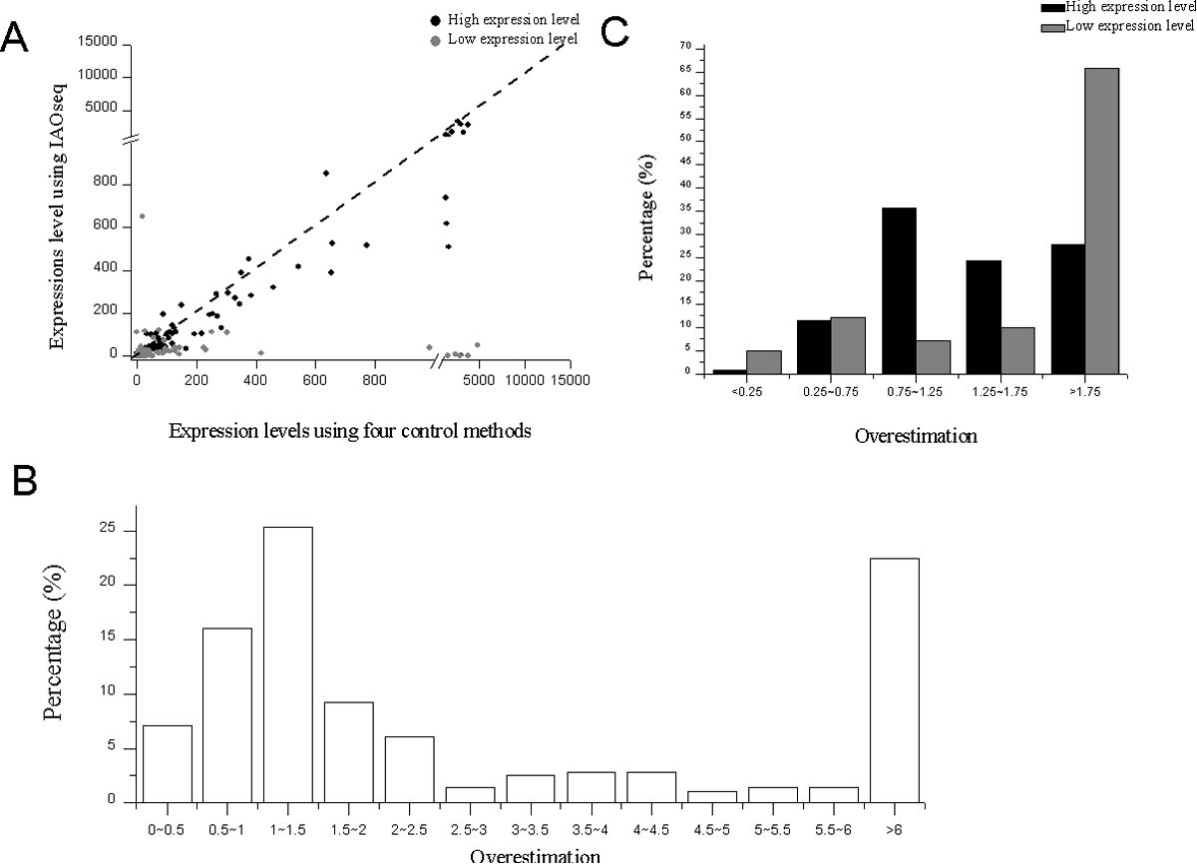
**Performances on same strand overlapping genes**. Scatterplot of the expression levels estimated by IAOseq and the average expression level estimated by other four methods from nonST data (A), overestimation on expression levels of all genes (B) and of genes in different levels of abundance by the commonly used methods (C). Overestimation is defined as the average expression level deduced by other four methods divided by the expression level deduced by IAOseq.

As transcripts from same strand overlapping genes have identical sequences, even the strand specific RNA-seq library construction method cannot distinguish from which gene template the transcripts were transcribed. It is reasonably that little difference was observed between the expression levels deduced from nonST data and from dUTP data using the five methods (Additional file 1: Figure S4). In contrast, IAOseq results showed that the expression levels of same strand overlapping genes were much lower than average abundance over the values estimated by the four methods (Cufflinks, RSEM, eXpress and Bitseq) (Figure 3A, Wilcoxon test, W = 29579, p-value = 5e-07). We estimated that the direct method for inferring gene expression levels gave an excessive overestimation of the expression levels of same strand overlapping genes with median of 1.61 (Figure 3B), and the overestimation is more obvious in genes with low expression levels (Figure 3C).

Our method could also be performed on the transcribed genomic regions covered by more than two overlapping genes with a mixture of overlapping types (Additional file 1: Figure S5).

### IAOseq performance on simulated data

As there are limited data from which to evaluate the accuracy of the quantification of overlapping gene expression, we further tested IAOseq on simulated data. More genes are excessive overestimated more than five folds by other five methods (Additional file 1: Figure S6A). Furthermore, for those overlapping genes which are simulated with no expression estimates, IAOseq show much better performance, more than 72% genes are estimated with low level, whereas overestimation is pronounced using other five methods (Additional file 1: Figure S6B).

## Conclusion

In summary, the output of this project provides a useful tool for inferring overlapping transcription levels, which aims to help us gain comprehensive understandings of the complex regulatory mechanism mediated by overlapping transcripts. IAOseq not only has a good performance on the adjustment of expression levels of different strand overlapping genes from nonST data, but also could be used to estimate expression levels of same strand overlapping genes, which is more interesting as most high-throughput protocols have the problem with same strand overlapping genes. IAOseq is as fast as other commonly used quantification methods. Overlapping expression is a universal feature of eukaryotic genomes and antisense mediated regulation could be an ancient mechanism to enhance gene expression response to genetic and environmental variation. In such scenario, the task of inferring expression levels of overlapping genes should be integrated into gene expression profile analysis.

## Availability

IAOseq is freely available at xxxx.

## List of abbreviations

nonST: non-strand specific RNA-seq dataset; LEARatio: the logarithm base 2 of estimated abundance ratio.

## Competing interests

The authors declare no conflict of interest.

## Authors' contributions

HS and YL designed the project and directed the analysis. SY and LT implemented the algorithm. SY performed the analysis. HS drafted the manuscript. All authors read and approved the final manuscript.

## Supplementary Material

Additional file 1 **This file contains Figures S1-S7 and Tables S1-S3**. Click here for file

## Declarations

The authors declare that funding for publication of the article was sponsored by Natural Science Foundation of China and the National '973' Basic Research Program.

This article has been published as part of *BMC Bioinformatics *Volume 16 Supplement 1, 2015: Selected articles from the Thirteenth Asia Pacific Bioinformatics Conference (APBC 2015): Bioinformatics. The full contents of the supplement are available online at http://www.biomedcentral.com/bmcbioinformatics/supplements/16/S1

## References

[B1] Cheng J Kapranov P Drenkow J Dike S Brubaker S Patel S Long J Stern D Tammana H Helt G Transcriptional maps of 10 human chromosomes at 5-nucleotide resolution Science 2005 308 5725 1149 1154 15790807 10.1126/science.1108625

[B2] Johnson JM Edwards S Shoemaker D Schadt EE Dark matter in the genome: evidence of widespread transcription detected by microarray tiling experiments Trends in Genetics 2005 21 2 93 102 15661355 10.1016/j.tig.2004.12.009

[B3] Sanna CR Li WH Zhang L Overlapping genes in the human and mouse genomes BMC genomics 2008 9 169 18410680 10.1186/1471-2164-9-169 PMC2335118

[B4] Chen J Sun M Kent WJ Huang X Xie H Wang W Zhou G Shi RZ Rowley JD Over 20% of human transcripts might form sense-antisense pairs Nucleic Acids Research 2004 32 16 4812 4820 15356298 10.1093/nar/gkh818 PMC519112

[B5] David L Huber W Granovskaia M Toedling J Palm CJ Bofkin L Jones T Davis RW Steinmetz LM A high-resolution map of transcription in the yeast genome Proc Natl Acad Sci USA 2006 103 14 5320 5325 16569694 10.1073/pnas.0601091103 PMC1414796

[B6] Sun M Hurst LD Carmichael GG Chen J Evidence for variation in abundance of antisense transcripts between multicellular animals but no relationship between antisense transcription and organismic complexity Genome Research 2006 16 7 922 933 16769979 10.1101/gr.5210006 PMC1484459

[B7] He Y Vogelstein B Velculescu VE Papadopoulos N Kinzler KW The antisense transcriptomes of human cells Science 2008 322 5909 1855 1857 19056939 10.1126/science.1163853 PMC2824178

[B8] Xu Z Wei W Gagneur J Perocchi F Clauder-Munster S Camblong J Guffanti E Stutz F Huber W Steinmetz LM Bidirectional promoters generate pervasive transcription in yeast Nature 2009 457 7232 1033 1037 19169243 10.1038/nature07728 PMC2766638

[B9] Wang XJ Gaasterland T Chua NH Genome-wide prediction and identification of cis-natural antisense transcripts in Arabidopsis thaliana Genome Biology 2005 6 4 R30 15833117 10.1186/gb-2005-6-4-r30 PMC1088958

[B10] Identification and analysis of functional elements in 1% of the human genome by the ENCODE pilot project Nature 2007 447 7146 799 816 17571346 10.1038/nature05874 PMC2212820

[B11] Carninci P Kasukawa T Katayama S Gough J Frith MC Maeda N Oyama R Ravasi T Lenhard B Wells C The transcriptional landscape of the mammalian genome Science 2005 309 5740 1559 1563 16141072 10.1126/science.1112014

[B12] Imanishi T Itoh T Suzuki Y O'Donovan C Fukuchi S Koyanagi KO Barrero RA Tamura T Yamaguchi-Kabata Y Tanino M Integrative annotation of 21,037 human genes validated by full-length cDNA clones PLoS Biol 2004 2 6 e162 15103394 10.1371/journal.pbio.0020162 PMC393292

[B13] Kapranov P Cheng J Dike S Nix DA Duttagupta R Willingham AT Stadler PF Hertel J Hackermüller J Hofacker IL RNA Maps Reveal New RNA Classes and a Possible Function for Pervasive Transcription Science 2007 316 5830 1484 1488 17510325 10.1126/science.1138341

[B14] Williams BA Slamovits CH Patron NJ Fast NM Keeling PJ A high frequency of overlapping gene expression in compacted eukaryotic genomes Proc Natl Acad Sci USA 2005 102 31 10936 10941 16037215 10.1073/pnas.0501321102 PMC1182411

[B15] Pinto S Michel C Schmidt-Glenewinkel H Harder N Rohr K Wild S Brors B Kyewski B Overlapping gene coexpression patterns in human medullary thymic epithelial cells generate self-antigen diversity Proc Natl Acad Sci USA 2013 110 37 E3497 3505 23980163 10.1073/pnas.1308311110 PMC3773787

[B16] Yukawa M Sugiura M Additional pathway to translate the downstream ndhK cistron in partially overlapping ndhC-ndhK mRNAs in chloroplasts Proc Natl Acad Sci USA 2013 110 14 5701 5706 23509265 10.1073/pnas.1219914110 PMC3619338

[B17] Tufarelli C Stanley JA Garrick D Sharpe JA Ayyub H Wood WG Higgs DR Transcription of antisense RNA leading to gene silencing and methylation as a novel cause of human genetic disease Nat Genet 2003 34 2 157 165 12730694 10.1038/ng1157

[B18] Yu W Gius D Onyango P Muldoon-Jacobs K Karp J Feinberg AP Cui H Epigenetic silencing of tumour suppressor gene p15 by its antisense RNA Nature 2008 451 7175 202 206 18185590 10.1038/nature06468 PMC2743558

[B19] Plagge A Non-coding RNAs at the Gnas and Snrpn-Ube3a imprinted gene loci and their involvement in hereditary disorders Frontiers in Genetics 2012 3 10.3389/fgene.2012.00264 PMC3509947 23226156

[B20] Group RGER, Group GS, Consortium tF Katayama S Tomaru Y Kasukawa T Waki K Nakanishi M Nakamura M Nishida H Antisense Transcription in the Mammalian Transcriptome Science 2005 309 5740 1564 1566 16141073 10.1126/science.1112009

[B21] Ge X Wu Q Jung YC Chen J Wang SM A large quantity of novel human antisense transcripts detected by LongSAGE Bioinformatics 2006 22 20 2475 2479 16895931 10.1093/bioinformatics/btl429

[B22] Harbers M Carninci P Tag-based approaches for transcriptome research and genome annotation Nat Methods 2005 2 7 495 502 15973418 10.1038/nmeth768

[B23] Metzker ML Sequencing technologies - the next generation Nat Rev Genet 2010 11 1 31 46 19997069 10.1038/nrg2626

[B24] Pelechano V Wei W Steinmetz LM Extensive transcriptional heterogeneity revealed by isoform profiling Nature 2013 497 7447 127 131 23615609 10.1038/nature12121 PMC3705217

[B25] Fullwood MJ Wei CL Liu ET Ruan Y Next-generation DNA sequencing of paired-end tags (PET) for transcriptome and genome analyses Genome Res 2009 19 4 521 532 19339662 10.1101/gr.074906.107 PMC3807531

[B26] Levin JZ Yassour M Adiconis X Nusbaum C Thompson DA Friedman N Gnirke A Regev A Comprehensive comparative analysis of strand-specific RNA sequencing methods Nat Methods 2010 7 9 709 715 20711195 10.1038/nmeth.1491 PMC3005310

[B27] Wang Z Gerstein M Snyder M RNA-Seq: a revolutionary tool for transcriptomics Nat Rev Genet 2009 10 1 57 63 19015660 10.1038/nrg2484 PMC2949280

[B28] Mangul S Caciula A Seesi SA Brinza D Banday AR Kanadia R An integer programming approach to novel transcript reconstruction from paired-end RNA-Seq reads Proceedings of the ACM Conference on Bioinformatics, Computational Biology and Biomedicine 2012 Orlando, Florida: ACM 369 376

[B29] Mezlini AM Smith EJ Fiume M Buske O Savich G Shah S Aparicion S Chiang D Goldenberg A Brudno M iReckon: Simultaneous isoform discovery and abundance estimation from RNA-seq data Genome Research 2012 10.1101/gr.142232.112 PMC3589540 23204306

[B30] Trapnell C Williams BA Pertea G Mortazavi A Kwan G van Baren MJ Salzberg SL Wold BJ Pachter L Transcript assembly and quantification by RNA-Seq reveals unannotated transcripts and isoform switching during cell differentiation Nature biotechnology 2010 28 5 511 515 20436464 10.1038/nbt.1621 PMC3146043

[B31] Yassour M Kaplan T Fraser HB Levin JZ Pfiffner J Adiconis X Schroth G Luo S Khrebtukova I Gnirke A Ab initio construction of a eukaryotic transcriptome by massively parallel mRNA sequencing Proc Natl Acad Sci USA 2009 106 9 3264 3269 19208812 10.1073/pnas.0812841106 PMC2638735

[B32] Guttman M Garber M Levin JZ Donaghey J Robinson J Adiconis X Fan L Koziol MJ Gnirke A Nusbaum C Ab initio reconstruction of cell type-specific transcriptomes in mouse reveals the conserved multi-exonic structure of lincRNAs Nat Biotechnol 2010 28 5 503 510 20436462 10.1038/nbt.1633 PMC2868100

[B33] Jiang H Wong WH Statistical inferences for isoform expression in RNA-Seq Bioinformatics 2009 25 8 1026 1032 19244387 10.1093/bioinformatics/btp113 PMC2666817

[B34] Mortazavi A Williams BA McCue K Schaeffer L Wold B Mapping and quantifying mammalian transcriptomes by RNA-Seq Nat Methods 2008 5 7 621 628 18516045 10.1038/nmeth.1226 PMC13303166

[B35] Wu Z Wang X Zhang X Using non-uniform read distribution models to improve isoform expression inference in RNA-Seq Bioinformatics 2011 27 4 502 508 21169371 10.1093/bioinformatics/btq696

[B36] Nagalakshmi U Wang Z Waern K Shou C Raha D Gerstein M Snyder M The transcriptional landscape of the yeast genome defined by RNA sequencing Science 2008 320 5881 1344 1349 18451266 10.1126/science.1158441 PMC2951732

[B37] Langmead B Trapnell C Pop M Salzberg SL Ultrafast and memory-efficient alignment of short DNA sequences to the human genome Genome Biol 2009 10 3 R25 19261174 10.1186/gb-2009-10-3-r25 PMC2690996

[B38] Li B Dewey C RSEM: accurate transcript quantification from RNA-Seq data with or without a reference genome BMC bioinformatics 2011 12 1 323 21816040 10.1186/1471-2105-12-323 PMC3163565

[B39] Cherry JM Hong EL Amundsen C Balakrishnan R Binkley G Chan ET Christie KR Costanzo MC Dwight SS Engel SR Saccharomyces Genome Database: the genomics resource of budding yeast Nucleic Acids Research 2012 40 D1 D700 D705 22110037 10.1093/nar/gkr1029 PMC3245034

[B40] Nicolae M Mangul S Mandoiu II Zelikovsky A Estimation of alternative splicing isoform frequencies from RNA-Seq data Algorithms for Molecular Biology 2011 6 1 9 21504602 10.1186/1748-7188-6-9 PMC3107792

[B41] Roberts A Pachter L Streaming fragment assignment for real-time analysis of sequencing experiments Nature methods 2012 10.1038/nmeth.2251 PMC3880119 23160280

[B42] Glaus P Honkela A Rattray M Identifying differentially expressed transcripts from RNA-seq data with biological variation Bioinformatics 2012 28 13 1721 1728 22563066 10.1093/bioinformatics/bts260 PMC3381971

