# Supplementary material for: IAOseq: inferring abundance of overlapping genes using RNA-seq data
Source: BMC Bioinformatics. 2015 Jan 21;16(Suppl 1):S3. doi: 10.1186/1471-2105-16-S1-S3 (PMC4331702; doi:10.1186/1471-2105-16-S1-S3)
Supplement: Additional file 1 — This file contains Figures S1-S7 and Tables S1-S3. [file 1471-2105-16-S1-S3-S1.doc]

**Supplementary Note**

To make use of the combination of the GBC and LBC, we let $b_{ij}=\alpha(G_{ij})+(1-\alpha)L_{ij}$. Supplementary table S1 summarizes the performances of IAOseq models with different parameter α settings. Letting α = 0 results in a model that incorporates only gene-specific read distributions. This model outputs a negative LEARatios, which means an underestimation of overlapping gene expression. And the other extreme, the IAOseq model with α = 1 shows the worse performance. From table S1, we can see that the IAOseq model with α = 0.1 achieves the smallest median LEARatios. Therefore, we chose α = 0.1 to illustrate the property of the IAOseq model.

**Supplementary Figures**


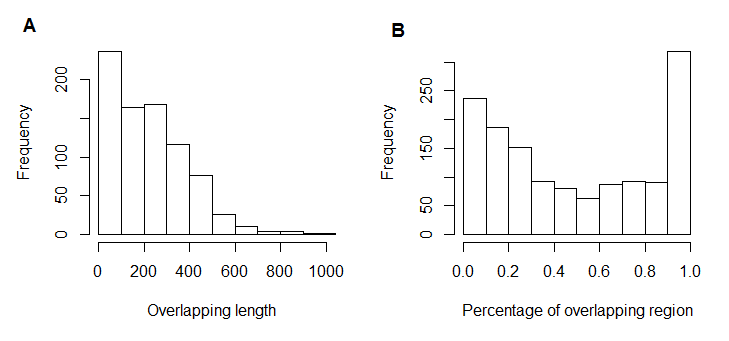


**Figure S1**: Overlapping length (bp) (A) and percentage of overlapping regions (B) for yeast overlapping genes.


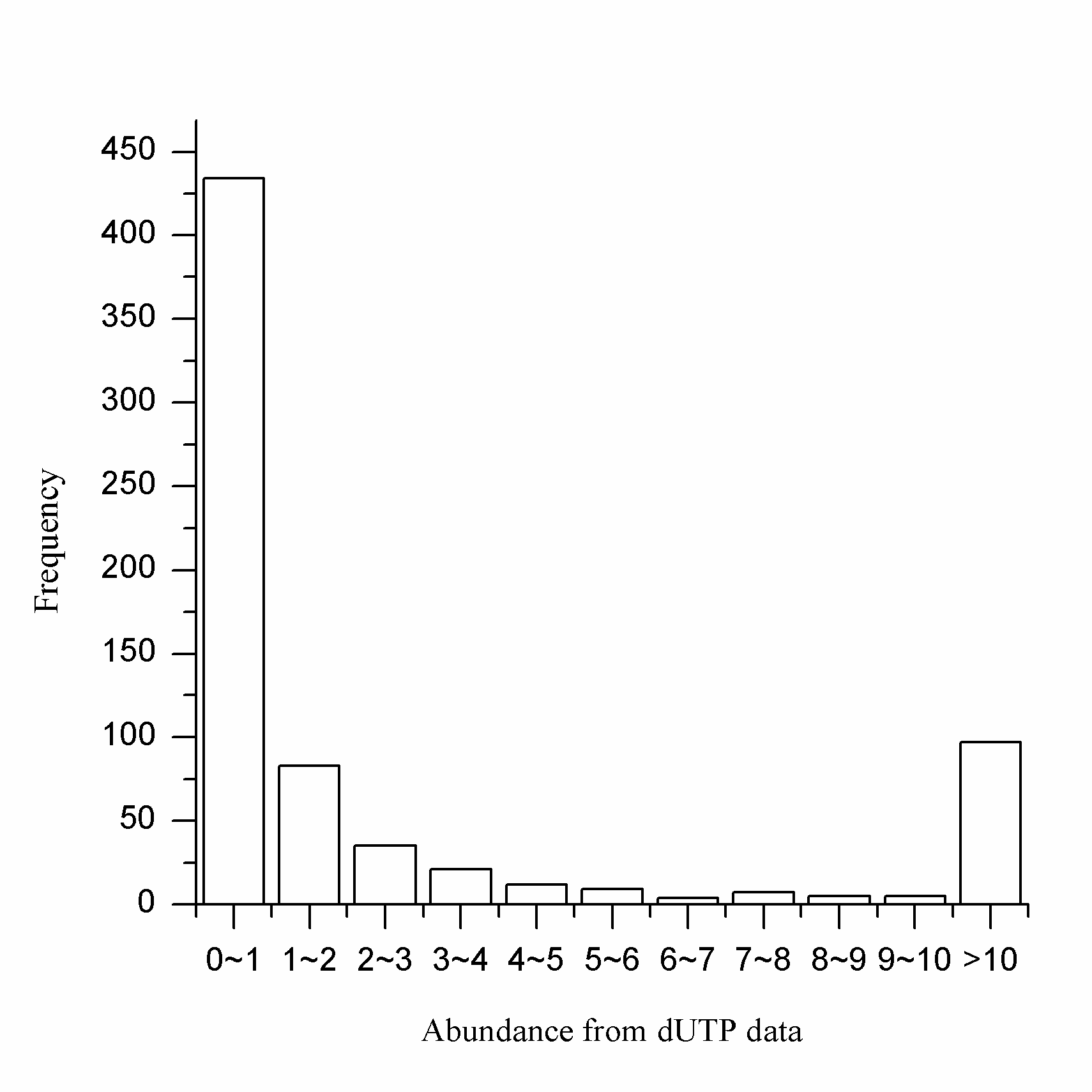


**Figure S2**: Transcriptional abundance of yeast dubious ORFs deduced from dUTP data.


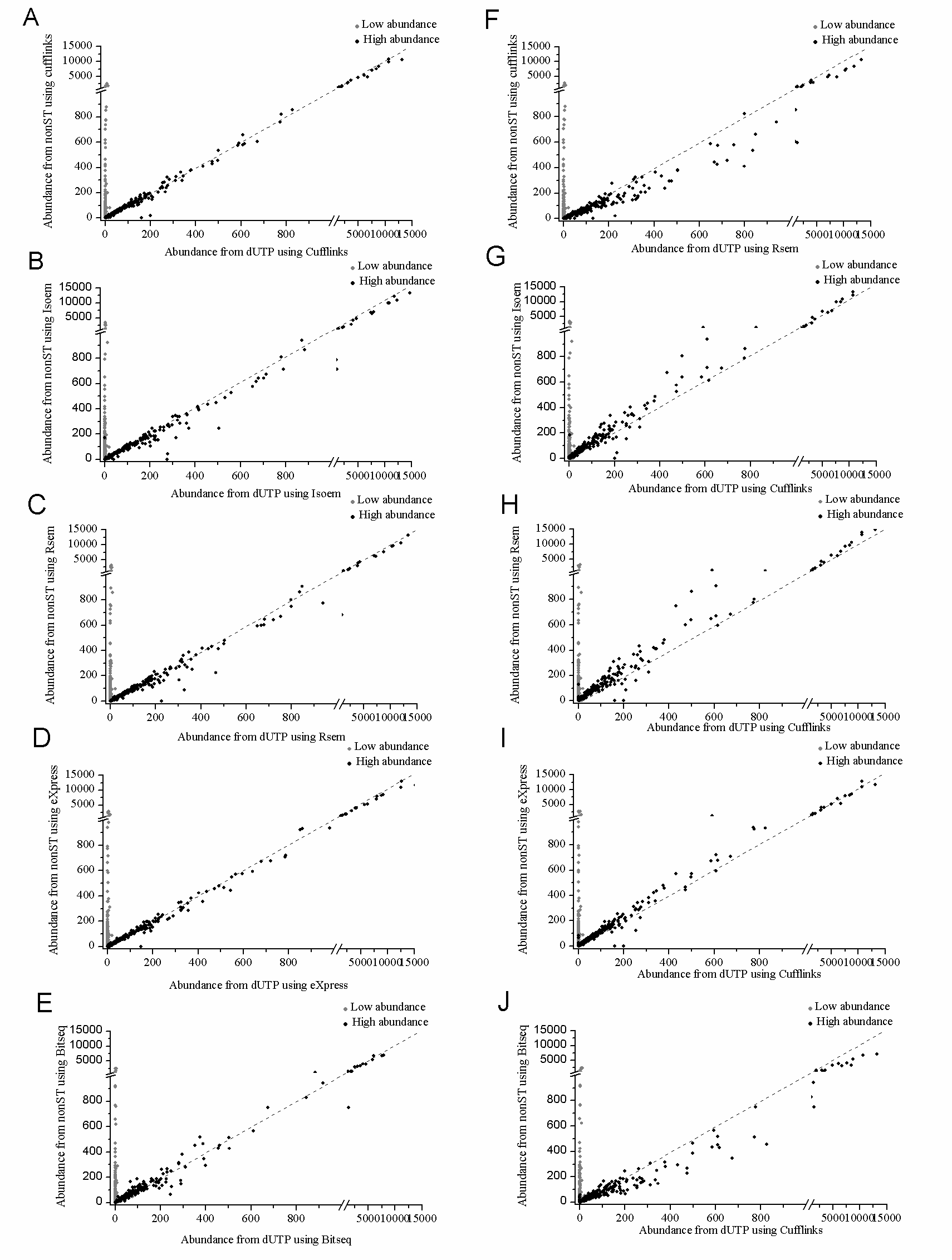
**Figure S3**: Scatterplot of the expression levels of different strand overlapping genes deduced from nonST and dUTP data using five other commonly used quantification methods, *i.e.* Cufflinks, Isoem, Resm, eXpress and Bitseq.

**
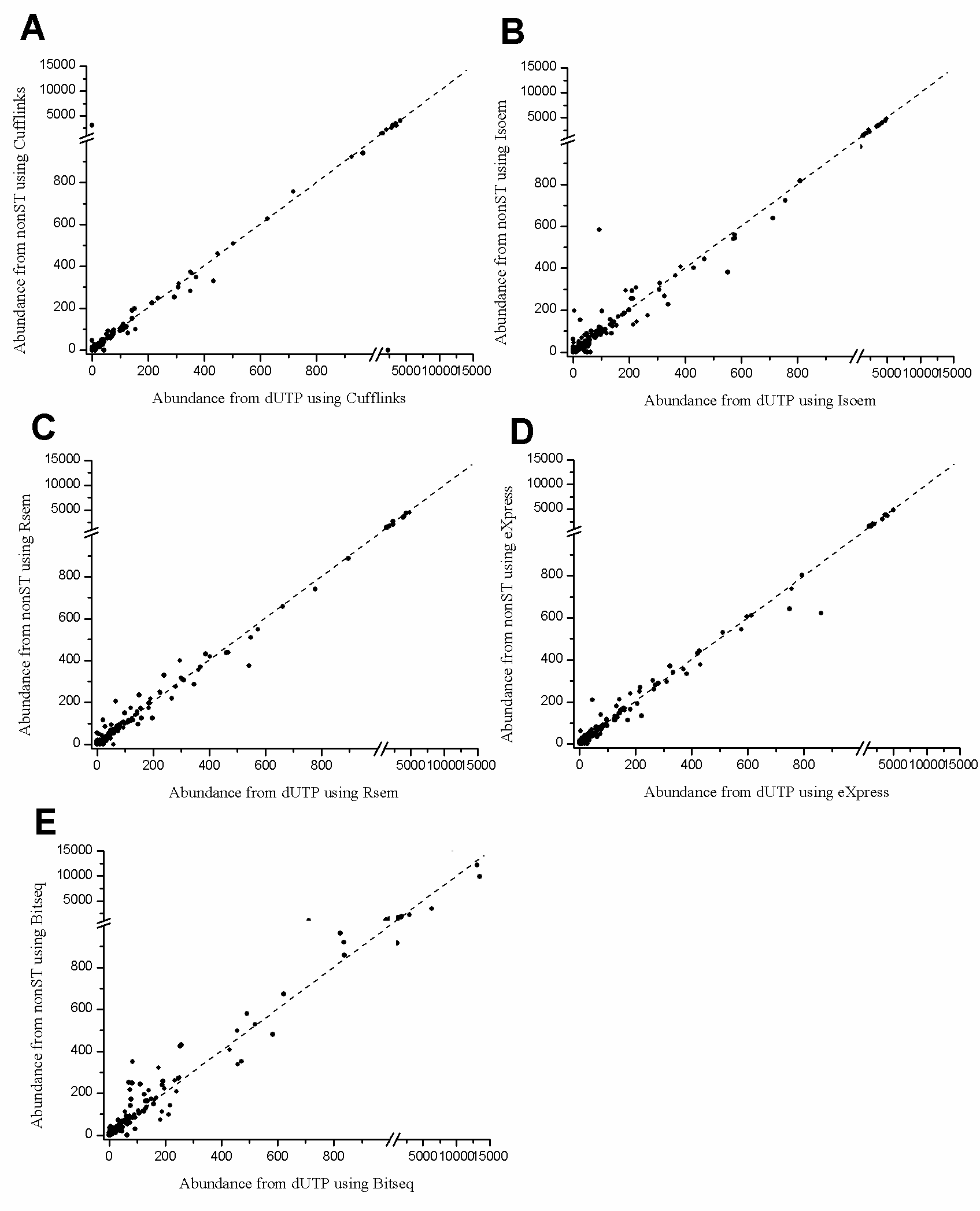
**

**Figure S4:** Scatterplot of the expression levels of same strand overlapping genes deduced from nonST and that deduced from dUTP data using five other commonly used quantification methods, *i.e.* Cufflinks, Isoem, Resm, eXpress and Bitseq.

**
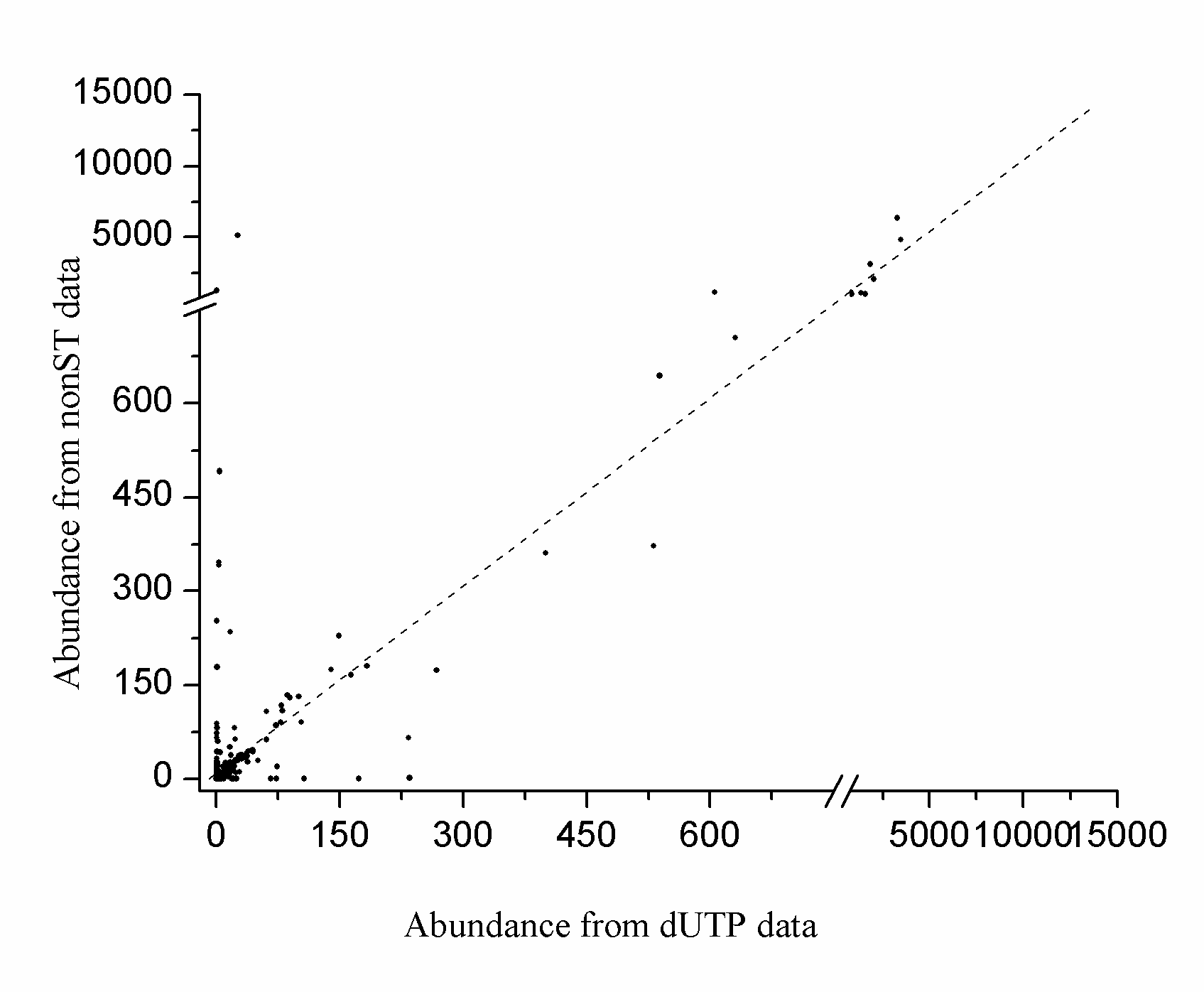
**

**Figure S5:** Scatterplot of the expression levels of multi-overlapping genes deduced from nonST and that deduced from dUTP data using IAOseq.


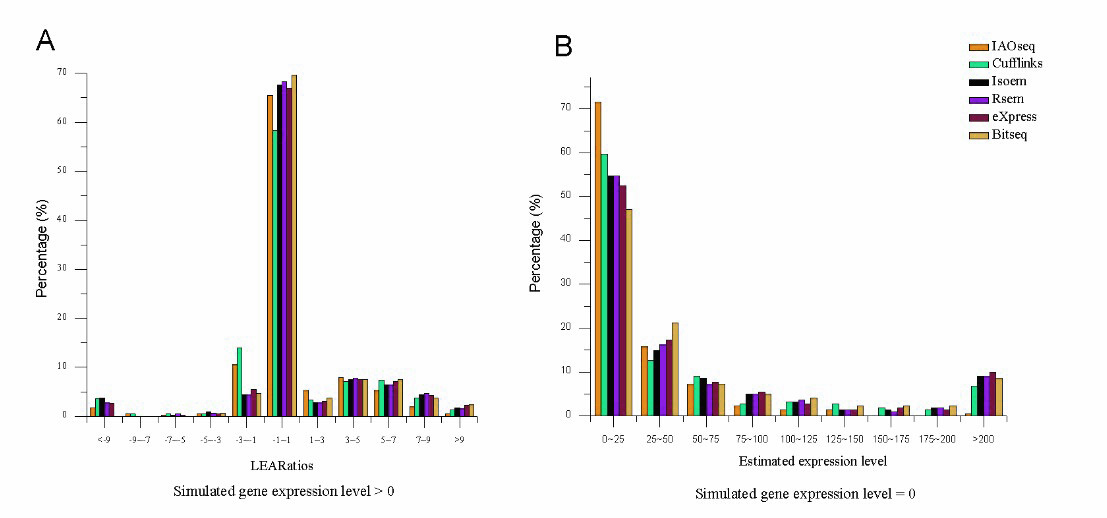


**Figure S6**: Performance of the six methods on simulated yeast data. (A) Percentage of genes within LEARatio intervals. (B) Percentage of genes simulated without expression within estimated abundance intervals.


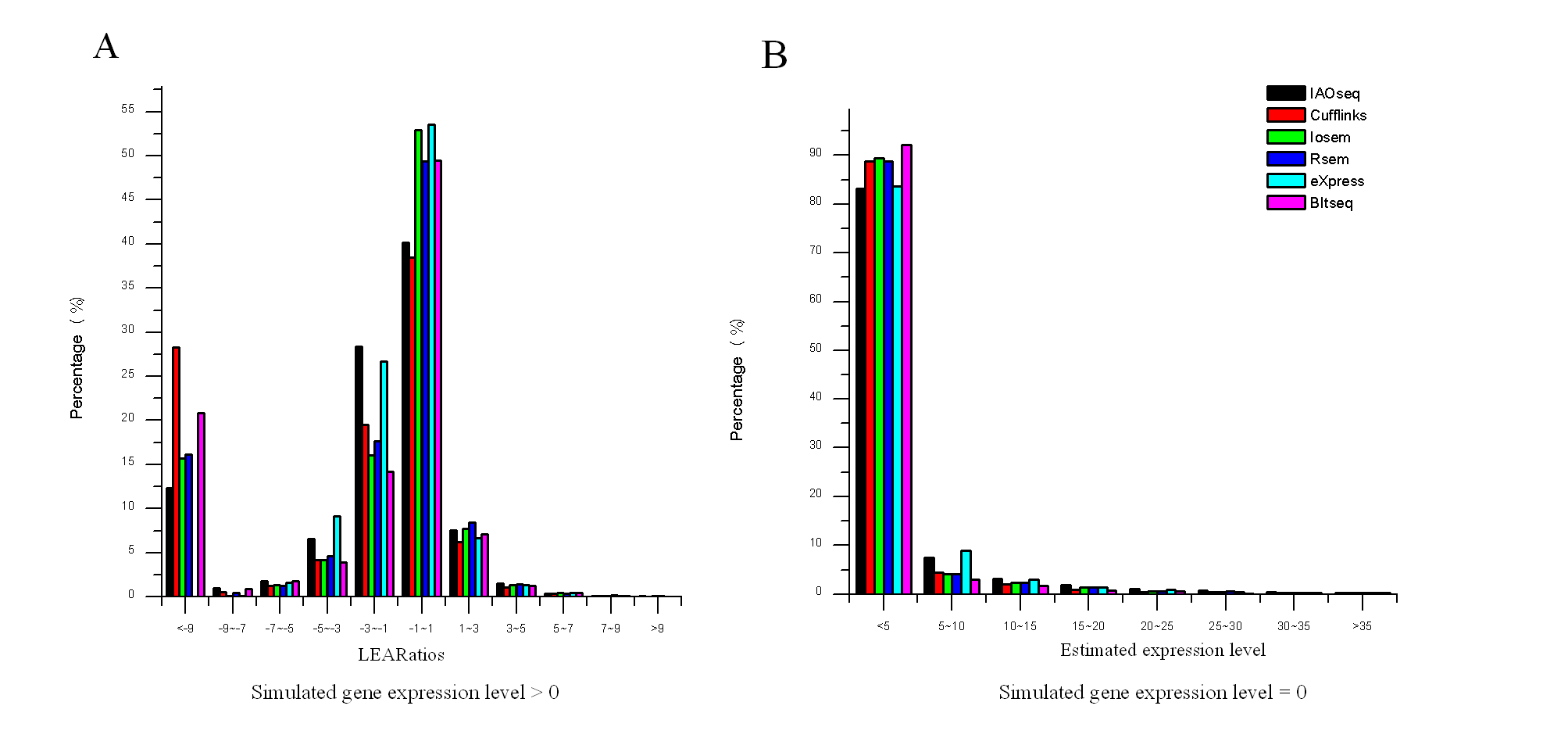


**Figure S7**: Performance of the six methods on simulated human data. (A) Percentage of genes within LEARatio intervals. (B) Percentage of genes simulated without expression within estimated abundance intervals.

**Supplementary Tables**

**Table S1**: Median LEARatios achieved by IAOseq models with differentαvalues.

| α | 0 | 0.1 | 0.2 | 0.3 | 0.4 | 0.5 | 0.6 | 0.7 | 0.8 | 0.9 | 1 |
| --- | --- | --- | --- | --- | --- | --- | --- | --- | --- | --- | --- |
| LEARatios | -0.0127 | 0.0469 | 0.0482 | 0.047 | 0.0596 | 0.0614 | 0.059 | 0.069 | 0.071 | 0.083 | 0.091 |

**Table S2**: Yeast overlapping genes.

| Overlapping type | Different strand overlapping | Same strand overlapping |
| --- | --- | --- |
| = 2 | 882 (12.3%) | 280 (3.9%) |
| ≥ 3 | 218 (3.1%) | 22 (0.3%) |
| Total | 1100 (13.7%) | 302 (4.2%) |

**Table S3**: Correlation between expression levels deduced from nonST and dUTP data.

|  | IAOseq | Cufflinks | Isoem | Rsem | eXpress | Bitseq |
| --- | --- | --- | --- | --- | --- | --- |
| R^2^ | 0.62 | 0.26 | 0.25 | 0.26 | 0.25 | 0.24 |
| P value | 2.2E-16 | 2.2E-16 | 2.2E-16 | 2.2E-16 | 2.2E-16 | 2.2E-16 |

Spearman's rho statistics were used to estimate the rank-based measure of associations.
